# The influence of progeny–Parents family travel on the well-being of the elderly in filial piety culture

**DOI:** 10.1371/journal.pone.0299565

**Published:** 2024-05-09

**Authors:** Yujia Wang, Xiong He, Fengying Zhang, Xiaoxia Zhang, Xiuying Hu, Xiaofeng Xie

**Affiliations:** 1 West China Hospital, Innovation Center of Nursing Research, and Nursing Key Laboratory of Sichuan Province, West China School of Nursing, Sichuan University, Chengdu, 610041, Sichuan Province, China; 2 Marxism College (Basic Department), Sichuan Water Conservancy Vocational College, Chengdu, 611830, Sichuan Province, China; 3 Department of General Surgery, Division of Breast Surgery, West China Hospital, Sichuan University; Breast Center, West China Hospital, Sichuan University, Chengdu, 610041, Sichuan Province, China; 4 Nursing Key Laboratory of Sichuan Province, Innovation Center of Nursing Research, Chengdu, 610041, Sichuan Province, China; Erasmus University Rotterdam, NETHERLANDS

## Abstract

Grounded in the cultural context of Chinese filial piety, this study employs structural equation model to analyze survey data from elderly participants. It explores the effect and path of progeny–parents family travel on the elderly’s sense of well-being and examines the mediating roles of generational interaction, optimistic emotion, and psychological resilience. The findings indicate that progeny–parents family travel positively influences the well-being of the elderly, with generational interaction, optimistic emotion, and psychological resilience serving as intermediary roles. Theoretically, this study enriches the localized perspective of family travel’s psychological and behavioral impact on the elderly. It elucidates the spillover effects of family travel within the framework of filial piety culture, delineates the mechanisms by which family travel enhances elderly well-being, and offers theoretical insights for businesses to develop customized family travel products and services.

## 1 Introduction

With China harboring the most rapidly expanding elderly demographic on the planet, a staggering one in five of its citizens is over 60, summing up to over 260 million individuals[1,2]. In response, the nation has embraced a robust strategy that places the well-being of its senior population at the forefront of state policy [3]. Strategic planning since the Eighteenth National Congress of the Communist Party has been instrumental in charting a future that not only ensures the elderly a life of health and contentment but also aligns with President Xi Jinping’s comprehensive vision for a flourishing society [4]. Well-being stands as a pivotal goal in life, particularly in the golden years. It mirrors the quality of life for seniors, acting as a barometer for successful aging [5]. At a personal level, well-being encapsulates the essence of “adding life to the years,” where the elderly find joy and satisfaction in the richness of their experiences. This state is a delicate interplay of subjective contentment and objective circumstances, showcasing the elderly’s zest to embrace their vitality [6]. Societally, bolstering seniors’ well-being serves as a buffer against the strains of a globally aging population and rising life expectancies [7]. Delving into the ways to enhance elderly well-being is not just beneficial but essential for fostering successful aging [8,9].

Travel is increasingly integral to daily life, particularly for the elderly, who are now a significant demographic in tourism, representing over 20% of tourists according to the National Committee on Aging. This shift reflects a broader trend where travel enhances the well-being and sense of fulfillment of older adults, fostering their adaptation to changing societal norms and contributing to a higher quality of life [10–14]. Beyond offering leisure, elderly tourism facilitates personal growth, allowing seniors to engage with new environments, which in turn rejuvenates their sense of vitality and fosters a unique connection with the world—a perspective that often eludes younger generations [15–17].

The principle of filial piety, deeply rooted in Chinese culture, not only shapes family dynamics but also reflects the societal norms experienced by the Chinese people [18]. This time-honored virtue, emphasizing respect for one’s elders, has long been the cornerstone of familial harmony and guides the interactions between generations [19]. In 1984, Xi Jinping authored an article in the People’s Daily titled “Middle-aged and Young Cadres should Respect the Elderly,” in which he revisited classical teachings to reinforce the virtue of respecting and caring for the elderly. The subject of how the elderly enjoy their twilight years and their ability to feel well-being is of concern not only to the older population but holds significant importance for younger generations as well [20]. In contemporary China, the well-being of the elderly, nurtured by social engagement and emotional well-being, is a testament to this tradition. Family activities, such as travel, have emerged as significant avenues for enriching the lives of the elderly, promoting joy, and fostering “successful aging,” a state of enriched life quality and satisfaction [21,22]. Intergenerational travel embodies the essence of filial piety, providing opportunities for emotional bonding and fulfilling the cultural expectations of familial duties [23,24].

The realm of family travel has been extensively examined in the literature, highlighting its multifaceted nature [25–27]. Despite the breadth of research, the specific dynamics of adult children journeying with their elderly parents is less charted [28]. Within the Chinese context, where the virtue of filial piety is deeply entrenched, such travel signifies more than leisure; it’s a profound act of kinship and duty. For adult children, these shared voyages are heartfelt gestures of reverence and care, deeply aligned with familial piety [26]. This trend has birthed a burgeoning segment in China’s tourism sector, underscored by a 2018 Ctrip survey, where a notable 54% of respondents prioritized traveling with their parents, a sentiment that topped the New Year’s wish list, reflecting the societal reverence for familial unity and intergenerational harmony [29]. Progeny–parents family travel, also known as “carrying the elderly” family travel, refers to a kind of travel mode in which two or three generations of families travel together. It not only reflects the traditional Chinese culture of filial piety, but also can effectively improve the emotional interaction between the elderly and their children and the quality of life and well-being of the elderly. Family travel is always described as a happy time, full of joy and well-being. Research indicates that family travel involving adult children and their elderly parents can positively impact the well-being of seniors throughout the travel experience [30–32] Anticipation of the journey often brings a sense of joy and excitement for both the elderly and their children [33]. During the trip, seniors can appreciate new environments, socially engage, and strengthen emotional bonds with their family, leading to a sense of relaxation and well-being. Post-travel, the rejuvenating effect continues as they reflect on their experiences, often reliving happy memories through photographs [31]. Therefore, the study of the influence mechanism of progeny–parents family travel on the well-being of the elderly under the traditional Chinese cultural concept of filial piety has important practical value in the fields of sociology and tourism marketing.

This research delves into the complex dynamics of how travel with adult offspring affects the well-being of the elderly within the Chinese filial piety framework. It sheds light on the roles of intergenerational engagement, positive emotional experiences, and the bolstering of psychological resilience in enhancing the life satisfaction of the elderly. The study offers robust empirical evidence supporting the positive impact of family travel on elderly well-being, thus addressing the broader societal issue of an aging population. The insights gleaned also carry significant implications for the refinement of tourism services and the crafting of bespoke travel experiences for families, enriching the field of tourism marketing. The research gap identified in this study pertains to the nuanced influence of intergenerational travel—specifically, travel involving adult children and their elderly parents—on the well-being of the elderly within the context of Chinese filial piety culture. While previous studies have examined family travel, there is a scarcity of empirical research focusing on the well-being effects of “caring the elderly” tourism from the perspective of filial duty and emotional bonds. This study aims to fill this gap by exploring how such travel activities can enhance the elderly’s life satisfaction and contribute to the successful aging paradigm in a society that highly values intergenerational relationships and filial responsibilities.

## 2. Literature review and research hypotheses

### 2.1 Literature review

#### 2.1.1 Progeny–parents family travel

As a common social phenomenon in the era of mass tourism, family travel started attracting widespread attention as early as the 1970s [27]. Family travel refers to activities that all or most family members can participate in, and its purpose is to enhance the affection between family members through tourism interaction. In terms of its classification, family travel can be interpreted in both a broad and narrow sense [34]. Family travel in the broad sense involves most family members, including parents (and parents-in-law), couples, children, and siblings, whereas family travel activities in the narrow sense include only the activities of couples and children (especially minor children). The core function of family travel is its positive effect and influence on family members [35]. Shin et al. [36], starting from the definition of family travel structure, intended to summarize the functional impact of family travel on individuals. The research results showed that family travel had a promoting effect on family interaction, relationship improvement, emotional establishment, and children’s cognition. In the opinion of Lyu et al. [37], the basic function of family travel is the emotional connection, but its deeper function is to promote communication and interaction between family members. Qiao et al. [38] believed that, compared with other types of family activities, family travel provides unique experiences for family members. On the one hand, it frees them from daily family life and gives them a different emotional experience. On the other hand, they can complete the construction from “I” to “we” through tourism interaction to consolidate existing family affection, further derive family identity and emotional belonging, and interpret family consciousness and the closeness of “us.” This study mainly focuses on the progeny–parents family travel activities of adult children and their parents and parents-in-law (hereinafter referred to collectively as parents).

Family travel is an important component of family happiness. Previous studies [35,39,40] have analyzed the relationship between family travel participation and family harmony, communication, satisfaction, and other family functions. However, the existing literature has mainly focused on family travel with adolescents, which largely ignores family travel between parents and adult children [41]. Progeny–parents family travel deserves more attention because the decisions and experiences are different from family travel with teenagers. Family travel where parents bring their teens along is often initiated by parents looking to make up for work absences in their children’s daily lives [27]. Family travel between adult children and their parents is usually initiated by adult children out of a desire to show filial piety [42]. They feel obligated to take their parents on a trip with their own money and free time when their parents are in good health. Fu et al. [43] proposed that the emotions established between parents and adult children through tourism and leisure may become a key part of family stability and parental happiness.

#### 2.1.2 Well-being of the elderly

Well-being was proposed by foreign psychologists in the middle of the 20th century and refers to the overall assessment of the quality of life of the evaluator according to certain standards. The concept later became widely used in the social sciences. Kardas et al. [44] proposed that well-being is composed of a feeling of life satisfaction and positive and negative emotions. At present, the research on the well-being of the elderly at home and abroad can be roughly divided into the two following categories: research on the influencing factors and research on the measurement methods. The influencing factors of well-being of the elderly include demographic factors, psychological factors, social economic factors, social support, social participation, and so on. Douka et al. [45] have shown that the well-being of the elderly is not significantly correlated with their age and gender; however, improving quality of sleep in the elderly could help boost their well-being. Seo [46] investigated the need for kinship among the elderly in South Korea and found that the difference between the expectation and satisfaction of the need for kinship between spouses and children significantly affected the well-being and psychological state of the elderly. Higher economic income can guarantee the material needs of the elderly, thus improving their well-being. There are great differences in the well-being of the elderly between urban and rural residents [47]. Marital status, gender, number of children, intergenerational relations, and healthcare connections are all significant factors that can impact the sense of well-being among the elderly. Moreover, in recent years, an increasing number of scholars have begun to pay attention to the impact of social participation on the welfare of senior citizens [48].

#### 2.1.3 Filial piety culture

As the ethical bedrock of traditional Chinese society, filial piety is integral to the familial structure and societal stability of China, serving as a moral compass for individual behavior [18]. Unlike the Western notion of family piety, which centers on “respect,” the traditional Chinese concept encompasses four tiers: benevolent acts towards parents, remembrance of their sacrifices, reverence for rulers, and pursuit of honor, all rooted in "respect" and "love." Since the 1960s, family evolution in post-developing countries has shown trends diverging from Western classical family theory predictions. This is particularly evident in China, where filial piety has adapted, balancing tradition with modernity [19]. Researchers suggest that traditional and modern conceptions of filial piety coexist within the social structure, shaping societal realities in tandem [49].

Amidst societal shifts, interpretations of filial piety inevitably evolve. The academic discourse frequently revisits how these values transition and integrate with modernity [50]. Traditional and modern filial piety continue to exert a profound influence on Chinese society, affecting both familial units and individuals. This cultural ethos is likely to inform family travel decisions, with adult children considering their parents’ needs as central to their planning. They seek to enhance parental well-being through travel, fulfilling their filial duties. The way adult children’s perceptions of filial piety influence travel choices and experiences, tailored to their parents’ desires, merits further exploration and analysis [20].

### 2.2 Theoretical basis and hypotheses development

#### 2.2.1 Theoretical basis

The appropriate theoretical foundation for the hypotheses presented in this study is rooted in a synthesis of the Activity Theory and the Social Support Theory [51]. The Activity Theory emphasizes the importance of ongoing social participation and leisure activities for the well-being of the elderly, suggesting that such engagement leads to improved life satisfaction and happiness [52]. The Social Support Theory underscores the role of family and friends in providing the emotional and practical support necessary for the elderly to cope with life’s challenges [53,54]. Building upon these frameworks, the proposed hypotheses explore how intergenerational family travel, underpinned by the cultural values of filial piety, impacts the psychological well-being of the elderly. The hypotheses posit that this form of travel not only fulfills familial duties but also reinforces emotional bonds, thereby enhancing the elderly’s sense of belonging, joy, and resilience. The dynamic interaction with adult children during travel is expected to lead to positive emotions and a strengthened psychological state, facilitating the elderly’s ability to navigate the complexities of aging with optimism and emotional stability. Building upon the aforementioned theories, this study clarifies the connections and interactions between Activity Theory and Social Support Theory. It demonstrates how these theories and values collectively enhance the psychological well-being of the elderly, accentuating key factors such as social participation, support from family and friends, and the resultant sense of belonging and emotional stability.

#### 2.2.2 Progeny–parents family travel and the elderly’s well-being

Family travel is a leisure and entertainment activity with a high participation of family members that has a profound impact on the psychology and emotions of each family member [55]. Its happiness effect has gradually become a social consensus and a hot research topic in the tourism field at home and abroad in recent years [56]. Relevant studies have explored the social attributes and happiness functions of family travel, and most of the research has verified that family travel activities have a positive impact on the well-being of family members to a certain extent. Family travel activities have a direct effect on increasing the well-being of tourists. At the same time, they can enhance the sense of well-being of family members by enhancing the emotional communication and intergenerational interaction of family members. Sie et al. [6] showed that travel activities have occupied a part of the lives of the elderly, and the level of travel activities has significantly increased the well-being of the elderly. This study has meticulously reviewed and synthesized both domestic and foreign literature to understand the multifaceted impact of travel activities on elderly well-being. The effect of progeny–parents family travel on the well-being of the elderly should be a dynamic process that lasts from pre-travel to the end of a tour. In the whole process of progeny–parents family travel, the psychological state of the elderly is a process of dynamic change of well-being, where the entire well-being evolution model is composed of well-being expectation, well-being experience, and well-being memory [57]. In this study, the effect and mechanism of progeny–parents family travel on the well-being of the elderly is explored in depth. By integrating a wide array of scholarly insights, this research delineates how family travel, particularly with adult children, contributes to the dynamic evolution of the elderly’s psychological state—from the anticipation and planning stages to the post-travel reflections.

#### 2.2.3 The mediating role of generational interaction

Family travel refers to activities in which all or most members of the family can participate in, and its purpose is to enhance affection between family members through tourism interaction [43]. As a specific social consumption mode, family travel plays a positive role in maintaining family relations, increases family harmony, enhances family common interests, and improves family member interactions and communications [58]. Family travel, as a new environmental stimulus, injects new vitality and happiness into the development of a family system [27]. Chinese filial piety culture attaches great importance to interpersonal interaction in the family context, emphasizing the children’s return to their parents’ parenting and teaching, and attaches importance to the children’s care for their parents based on the needs and preferences of their parents [43]. Moreover, filial piety also attaches importance to parent–child filial piety between parents and adult children, that is, it emphasizes mutual care between parents and adult children, emotional interaction between parents and adult children, and equality in the emotional interaction. In the process of progeny–parents family travel, adult children’s choices and decisions on travel destinations, travel modes, and accommodations are mostly based on their parents’ travel needs, so that parents can experience convenience and pleasure, thereby promoting family emotional interaction [35]. Therefore, because of the influence of filial piety culture, when children travel together with their parents, they consider their parents as the persons of honor and prioritize the parents’ travel demands throughout the entire family travel decision-making process. Likewise, parents indulge their own requirements to attain the highest possible level of satisfaction [28]. This allows for the realization of emotional communications and a benign interaction between children and parents during the entire tourism experience. Hence, this paper puts forward the following hypothesis:

*H1*: *Progeny–parents family travel positively affects generational interaction between parents and adult children*.

According to the activity theory, family activities are the basis of social life, and the elderly who actively participate in family activities have a higher degree of happiness [59]. The changes in the social roles of the elderly (e.g., from the labor role to the idle role, from the main role to the dependent role, and from the spouse role to the single role) narrow the scope of their participation in activities and make them prone to loneliness, emptiness, depression, and other negative emotions [5,46]. Generational interaction and travel experience during family travel can increase the elderly’s social participation and mitigate and weaken the social and psychological discomfort caused by such role changes, thus increasing the elderly’s sense of well-being [60]. In the process of travel, the company of and communication with their adult children can make the elderly feel satisfied during the trip. Constraints in the interpersonal category, such as intergenerational conflict, are thought to be of primary concern to the elderly when making travel decisions. Therefore, generational interaction in family travel is an important reason for the elderly to participate in tourism activities and obtain happiness. Such generational interaction plays a mediating role between progeny–parents family travel and the well-being of the elderly. Therefore, this paper postulates the following:

*H2*: *Generational interaction significantly affects the well-being of the elderly*.

#### 2.2.4 The mediating role of optimistic emotions

According to the social support theory, the social support obtained by individuals usually comes from family and friends, which is manifested as timely feedback and communication, active support, listening, and encouragement [61]. Social support plays an important role in relieving pressure and creating positive emotions in the elderly. Panebianco et al. [62] and Sun et al. [63] have shown that most negative emotions experienced by the elderly come from the shrinking of social networks, decreasing frequency of social interaction, and decreasing degree of social support. Family members’ support is one of the most powerful and indispensable types of social support for the elderly. Stronger family ties were associated with higher levels of positive emotions and life satisfaction in the elderly [41]. As a way for the elderly to enrich their spirituality and pursue a happy life, family travel has become a popular activity that children are willing to support their parents participating in [27]. Adult children also regard family travel to strengthen their connection with their parents, strengthen the link between parents and the society, express filial piety, and make up for a lack of time with their parents [43]. Family travel not only helps the elderly to build positive social relations, but also promotes the elderly’s emotional improvement and spiritual development. In China, family travel provides many positive psychological needs for the elderly, elevates their happiness, and reflects the transformation of the dedicated family concept of the contemporary elderly. Hence, this paper proposes the following hypothesis:

*H3*: *Progeny–parents family travel positively affects the optimistic emotions of the elderly*.

Positive psychology believes that positive mental states such as optimism and tenacity are an important endogenous factor affecting individual behavior and life satisfaction [44]. Compared with short-term emotions (e.g., happiness, sorrow, and joy) and stable qualities (e.g., personality and temperament), positive emotions are relatively stable and malleable, easy to cultivate and develop, and are an important endogenous force to guide the elderly to be positive and experience happiness. Compared with other stages of life, loss and trauma in old age are more common and fatal [45]. The quality of life of the elderly is seriously affected by the decline of physical function, the breakdown of social relationships, and the gradual deaths of loved ones [64]. It is more necessary for the elderly to have key positive emotions such as optimistic emotion and resilience to cope with loss and rebuild a happy life. Relevant studies have found that, compared with young people, non-structural constraints such as psychological and emotional states are more likely to be the main obstacles affecting the happiness and life satisfaction of the elderly.

The more positive the elderly are and the more they looked forward to the future, the greater their level of happiness [47]. In family travel, adult children’s company and support for parents can strengthen family bonds. Under such circumstances, parent–child relationships tend to be harmonious, with positive emotions being stimulated in the elderly, thereby enhancing the subjective well-being of the elderly [43]. Therefore, family travel is an effective way for families, especially adult children, to support the elderly to achieve positive psychology, happiness, and quality of life [58]. Optimistic emotions play a mediating role between progeny–parents family travel and the well-being of the elderly. Based on the above analysis, we propose the following hypothesis:

*H4*: *Optimistic emotions significantly improve the well-being of the elderly*.

#### 2.2.5 The mediating role of psychological resilience

Psychological resilience refers to a kind of psychological strength that everyone possesses [65]. It is a process of effectively adapting to and managing stress or trauma, which can moderate the negative effects of stress and enhance the confidence and courage of individuals to cope with difficulties. Enhancing self-exploration of the environment and the unknown during travel is an important part of the psychological resilience of the elderly and is a way to actively participate in social activities. Going places that one has never been to and viewing scenery that one has never seen before having become the dream of many elderly people [66].

The function of family travel in improving the psychological resilience of the elderly and enhancing family cohesion has been verified in many families travel studies at home and abroad [41]. Psychological resilience and happiness-boosting effects have also been identified by scholars as positive effects of family travel on the elderly and the family [55]. On the one hand, progeny–parents family travel can help elderly parents expand their horizons, update their cognition, and learn new knowledge and skills [43]. On the other hand, it also helps to teach children to respect the old and love the young and acquire the skills and art of intergenerational communication. Research has shown that, after family travel, parents think highly of the travel experience, feel it is enjoyable to be with family, and have a strong desire to travel again with their children. Parents either selectively ignore or downplay the unpleasant experiences during tourism, showing strong intergenerational emotional inclinations [29]. In our study, many of the elderly said that deciding to travel with their children was not important; in their view, the important thing is to have the opportunity to be with their children, to experience filial piety, to feel a sense of ceremony, to feel their children are capable, to feel blessed, and to let others feel envy [58]. Psychological resilience reflects the long-term impact of progeny–parents family travel on the positive mental state of the elderly. Hence, this study postulates the following:

*H5*: *Progeny–parents family travel significantly affects the psychological resilience of the elderly*.

With an increase in age, the physical and cognitive functions of the elderly show a downward trend [67]. However, individual emotions do not necessarily present a downward trend. Studies have shown that the elderly show a high level of emotional control, emotional stability, and emotional maturity, and the regulation and balance of positive emotions can effectively reduce anxiety, depression, stress, and other negative emotions. Blanchflower [68] research found that the age distribution of well-being decreased first and then increased, and the elderly may have a stronger sense of well-being than the young. Even when older people face declining health, illness, and social disengagement from retirement, their well-being is not necessarily negatively affected [69]. In contrast, well-being may remain high in older people. Psychological resilience can help the elderly to accept, deal with, and adjust to various adverse conditions such as physical function decline, cognitive decline, social status decline, income reduction, and so forth [70]. The elderly who display strong psychological resilience are more able to maintain physical and mental health, obtain happiness, achieve social adaptation, and enjoy successful aging. Psychological resilience plays a mediating role between progeny–parents family travel and the well-being of the elderly. Hence, this study proposes the following hypothesis:

*H6*: *Psychological resilience significantly affects the well-being of the elderly*.

According to the above analysis, positive emotional communication and intergenerational interaction between parents and adult children not only help to create a warm and harmonious family atmosphere, but also significantly improve the positive psychological mood and mental health of the elderly [43]. In addition, the personality theory suggests that a happy person is not only able to experience more positive emotions and less negative emotions but is also more likely to experience the positive and resilient side of life [69]. When elders experience positive emotions, their psychological resilience is bolstered, equipping them to more effectively navigate the challenges associated with aging, such as the decline in physical abilities, social disengagement, and various other hardships [67]. Thus, optimistic emotions play an important role in improving the happiness of the elderly. Therefore, this study proposes the following two hypotheses:

*H7*: *Generational interaction significantly affects the optimistic emotions of the elderly*.
*H8*: *Optimistic emotions significantly affect the psychological resilience of the elderly*.

**Fig 1:**
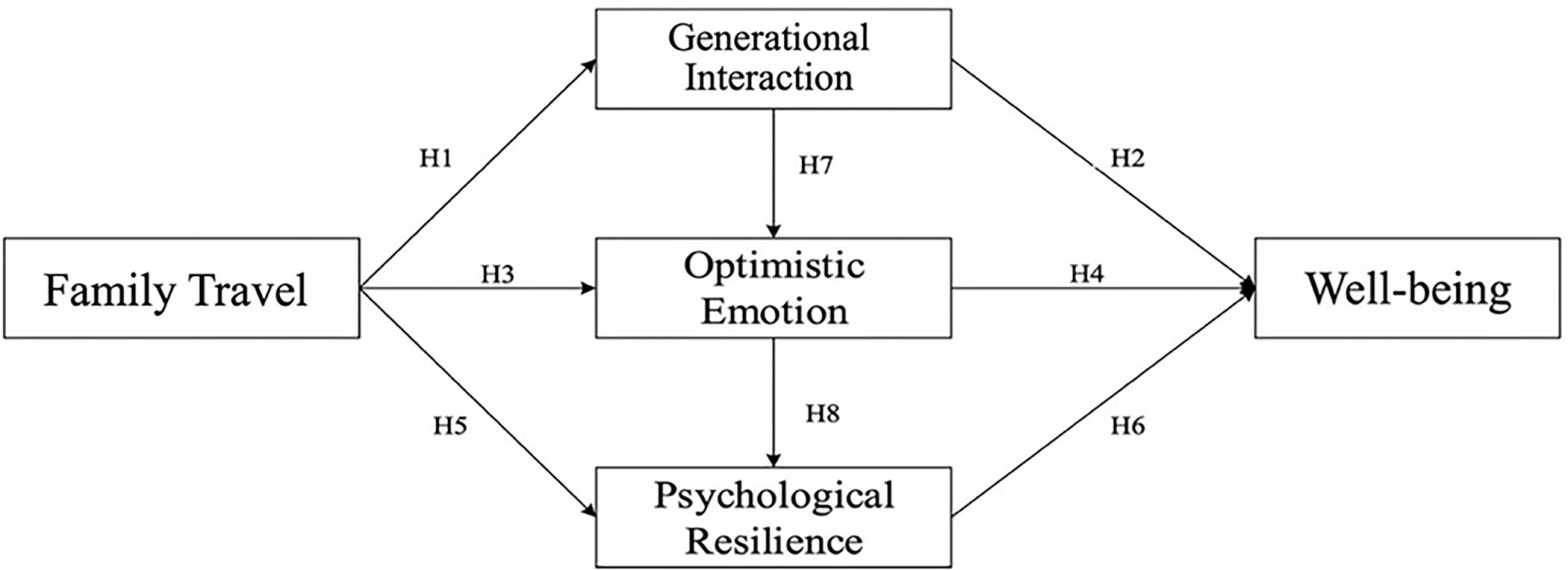
Hypothesized theoretical model.

Based on the concept of filial piety culture, progeny–parents family travel, the elderly’s well-being, and the relevant literature, a conceptual framework has been constructed as shown in Fig 1.

## 3 Methodology

### 3.1 Sample and data collection

The purpose of this study is to explore the influence of progeny–parents family travel on the well-being of the elderly through real data. In this paper, the validated scale was used to prepare the questionnaire after appropriate adjustment according to the research situation. The questionnaires were distributed to the subjects through sojump (China’s largest online survey platform), the reliability and validity of the collected valid data were tested, and all the hypotheses of the study were verified through structural equation modeling (SEM).

According to the research topic, the elderly over 60 years old were selected as Subjects. Participants were recruited from July 10, 2022 to December 28, 2022. This study was approved by the Medical Ethics Committee (No. 2022–0787) of West China Hospital of Sichuan University. Written informed consent was obtained from all participants. The questionnaires were distributed on Sojump. A total of 350 questionnaires were received, and questionnaires with extremely short answer times or obvious regularity were excluded. In total, 323 valid questionnaires were obtained for a valid recovery rate of 92.29%.

### 3.2 Measurement items of variables

The measurement of well-being in the elderly includes the assessment of overall well-being, which is more commonly used in social and psychological studies [57,71]. Some scholars have studied the well-being of the elderly based on CHARLS, CGSS, CLHLS, CFPS, and other databases [72,73]. In these studies, researchers used a holistic approach, asking people to rate their happiness based on their true thoughts and feelings. Another method is to measure the well-being of the elderly using a well-being assessment scale, among which the Memorial University of Newfoundland Well-being Scale (MUNSH) is the most widely used [74,75]. Zhang (2020) used the MUNSH scale to measure the subjective well-being of the elderly.

All items were measured using existing, reliable scales with adequate internal consistency, composite reliability, and convergent validity. To measure progeny–parents family travel, three items developed by Fu and Zhang [43] and Sie et al. [6] were used (i.e., “You like to travel,” “You and your family often travel together,” and “You and your family have traveled to many places”). To measure generational interaction in filial piety culture, four items developed by Bai et al. [76] and Li and Chan[21] were used (e.g., “You usually help your children look after their children or do housework,” “You think that children taking their parents to travel shows filial piety and repayment,” and “You think children will pay special attention to taking care of and obeying their parents’ wishes when travelling with their parents”). To measure optimistic emotions, three items developed by Bai et al. [76] and Fu et al.[29] were used (i.e., “When you travel with your children, your relationship with them becomes more harmonious and closer,” “When you travel with your family, you feel that you have broadened your horizons and learned more about local customs,” and “When you travel with your family, you can adapt to the local eating habits and climate”). To measure psychological resilience, four items developed by Koenen [65] and Ye and Zhang [77] were used (e.g., “You are very impressed by the experience of traveling with your family,” “When you travel with your family, you feel young, full, and happy,” and “You are looking forward to traveling with your family in the future”). To measure well-being, five items developed by Hendriks et al. [71] and Smith et al. [51]were used, which were modified according to the research situation (e.g., “You are particularly satisfied with your present life,” “you think you are in a good mood,” and “you are happy and satisfied with your present life”). All the measures were scored on a five-point Likert scale (1 = strongly disagree to 5 = strongly agree). The measurement items are presented in Table 1.

**Table 1:** Measurement items.

Constructs	Code	Items	Source
Family Travel (FT)	FT1	You like to travel	Fu and Zhang[29]; Sie et al. [6]
	FT2	You and your family often travel together	
	FT3	You and your family have traveled to many places	
Generational Interaction (GI)	GI1	You usually help your children look after children or do housework	Bai et al. [75]; Li and Chan [13]
	GI2	You think that children taking their parents to travel shows filial piety and repayment	
	GI3	You think children will pay special attention to, take care of and obey their parents’ wishes when travel with their parents	
	GI4	When chatting with others, you will specifically mention the relevant experience and feelings of traveling with your children	
Optimistic Emotion (OE)	OE1	When you travel with your children, your relationship with them becomes more harmonious and closer	Bai et al. [76]; Fu et al. [29]
	OE2	When you travel with your family, you feel that you have broadened your horizons and learned more about local customs	
	OE3	When you travel with your family, you can adapt to the local eating habits and climate	
Psychological Resilience (PR)	PR1	You are very impressed by the experience of traveling with your family	Denckla [65]; Ye and Zhang [77]
	PR2	When you travel with your family, you feel young, full, and happy	
	PR3	You are looking forward to traveling with your family in the future	
	PR4	Travel experience increases your self-confidence, optimism, and interpersonal skills	
Well-being (WB)	WB1	You are particularly satisfied with your present life	Hendriks et al. [71]; Smith et al. [57]
	WB2	you think you are in a good mood	
	WB3	you are happy and satisfied with your present life	
	WB4	You cherish your present life	
	WB5	Compared with your peers, your health status is similar or even better	

## 4 Results

### 4.1 Descriptive statistical analysis

The demographic indicators include gender, age, occupation before retirement, educational level, monthly income (in CNY), and who the respondents live with. The results of the descriptive statistical analysis indicated that 51.42% of the respondents were male and 48.58% were female. Of the participants, 55.42% were between 60–69 years old, 34.67% participants were between 70–79 years old, and 9.91% participants were over 80 years old. For occupations before retirement, public officials accounted for 32.31%, medical workers accounted for 9.43%, other professional technicians accounted for 12.97%, self-employed workers accounted for 33.02%, the category “other” accounted for 4.72%, and unemployed accounted for 7.55%. Of the participants, 67.22% had a high school degree or below, 27.36% had a college/university degree, and 5.42% had a master’s or doctoral degree. Of the participants, 56.36% had a monthly income under CNY 5,000, 24.57% had a monthly income ranging from CNY 5,000–8,000, 12.03% had a monthly income ranging from CNY 8,001–10,000, and 7.54% had a monthly income of over CNY 10,001. The demographic characteristics are presented in Table 2.

**Table 2:** Demographic characteristics.

Characteristics	Number	Percentage (%)
**Gender**		
Male	218	51.42%
Female	206	48.58%
**Age**		
60–69	235	55.42%
70–79	147	34.67%
Over 80	42	9.91%
**Occupation**		
Public officials	137	32.31%
Medical workers	40	9.43%
Other professional technicians	55	12.97%
Self-employed	140	33.02%
Other	20	4.72%
Unemployed	32	7.55%
**Education Level**		
High school or below	285	67.22%
College/University	116	27.36%
Master or doctoral	23	5.42%
**Income (Monthly, CNY)**		
<5000CNY	239	56.36%
5001 CNY—8000 CNY	102	24.57%
8001 CNY—10,000 CNY	51	12.03%
>10,001 CNY	32	7.54%
**Live with**		
Children	120	28.30%
Spouse	138	32.55%
Spouse and children	114	26.89%
Alone	31	7.31%
Other	21	4.95%

### 4.2 Common method bias

A single-factor test was conducted to examine common method bias in accordance with Harman (1967). The results showed that the largest eigenvalue explained 38.4% variance (below 50%), suggesting that there was no common method bias in the collected data[78].

Because the survey questionnaire in this study is self-reported, it may cause common method bias. This paper designed a questionnaire based on procedural remedies proposed by Tehseen et al. [79] to control such deviations. First, all items in the questionnaire were expressed succinctly to avoid vague expressions. Second, multiple items were used to measure each variable to eliminate the effect of participants discerning the relationship between the study’s purpose and the variables. In addition, the anonymity of the participants was ensured, the participants were told that there was no right or wrong answer for each item, and that there was no difference between high and low scores.

### 4.3 Reliability analysis

A model is considered reliable when the Cronbach’s α and composite reliability of each latent construct are equal to or greater than 0.8 [80,81]. In this study, Cronbach’s α and composite reliability range from 0.904 to 0.951, exceeding the threshold value of 0.8. SPSS 22.0 was used to test the reliability of the scales, and the results showed that the Cronbach’s α of family travel is 0.904, the Cronbach’s α of generational interaction (filial piety) is 0.914, the Cronbach’s α of optimistic emotion is 0.919, the Cronbach’s α of psychological resilience is 0.915, and the Cronbach’s α of well-being is 0.951. All the values of Cronbach’s α are greater than 0.8, which proves that these scales have a high degree of reliability. The means, SDs, and Cronbach’s α results are presented in Table 3.

**Table 3:** Means, SD, Cronbach’α, CR and AVE.

Variables	Mean	SD	Cronbach’ α	AVE	CR	1	2	3	4	5
1. Family Travel	5.35	0.906	0.904	0.759	0.904	[0.871]				
2. Generational Interaction	5.32	0.754	0.914	0.730	0.915	0.623	[0.854]			
3. Optimistic Emotion	5.29	0.914	0.919	0.792	0.919	0.619	0.480	[0.890]		
4. Psychological Resilience	5.13	0.79	0.915	0.727	0.914	0.618	0.605	0.492	[0.853]	
5. Well-being	5.32	0.909	0.951	0.796	0.951	0.660	0.626	0.503	0.528	[0.892]

Note: N = 424; SD = standard deviation; CR = composite reliability; AVE = average variance extracted; All correlations are significant at the 0.01 level. [] is the square root of AVE.

### 4.4 Confirmatory factor analysis

Because all the scales used in this study are validated scales that have been widely used and verified, they can be considered to have good content validity. Confirmatory factor analysis of all sample data was performed by AMOS 22.0 software, and the result showed that the model fitted well (*χ*^*2*^ = 235.323, *df* = 142, *χ*^*2*^*/df* = 1.657 < 3, RMSEA = 0.039 < 0.08, CFI = 0.977 > 0.9, NFI = 0.968 > 0.9). The standardized factor loadings of all items ranged from 0.819 to 0.911 and all values were statistically significant, indicating that the measured items adequately represent and reflect the dimension content in which they are located (see Table 4). The correlation coefficients among all constructs were always less than the square root of the mean extracted variance, indicating that the model had high discriminant validity. In conclusion, all scales have good reliability and validity.

**Table 4:** Results of confirmatory factor analysis.

Variables	Code	Items	Factor Loading
Family Travel (FT)	FT1	You like to travel	0.869
	FT2	You and your family often travel together	0.831
	FT3	You and your family have traveled to many places	0.911
Generational Interaction (GI)	GI1	You usually help your children look after children or do housework	0.862
	GI2	You think that children taking their parents to travel shows filial piety and repayment	0.839
	GI3	You think children will pay special attention to take care of and obey their parents’ wishes when travel with their parents	0.819
	GI4	When chatting with others, you will specifically mention the relevant experience and feelings of traveling with your children	0.889
Optimistic Emotion (OE)	OE1	When you travel with your children, your relationship with them becomes more harmonious and closer	0.897
	OE2	When you travel with your family, you feel that you have broadened your horizons and learned more about local customs	0.891
	OE3	When you travel with your family, you can adapt to the local eating habits and climate	0.881
Psychological Resilience (PR)	PR1	You are very impressed by the experience of traveling with your family	0.859
	PR2	When you travel with your family, you feel young, full, and happy	0.849
	PR3	You are looking forward to traveling with your family in the future	0.839
	PR4	Travel experience increases your self-confidence, optimism, and interpersonal skills	0.870
Well-being (WB)	WB1	You are particularly satisfied with your present life	0.895
	WB2	you think you are in a good mood	0.896
	WB3	you are happy and satisfied with your present life	0.877
	WB4	You cherish your present life	0.903
	WB5	Compared with your peers, your health status is similar or even better	0.890

### 4.5 Structural model evaluation

Using the maximum likelihood estimation method, SEM was conducted to examine the hypotheses. The results are shown in Table 5. The overall fit of the structural model was as follows: *χ*^*2*^ = 302.336, *df* = 144, *χ*^*2*^*/df* = 2.100 < 3, RMSEA = 0.051 < 0.08, GFI = 0.931 > 0.85, CFI = 0.978 > 0.9, NFI = 0.958 > 0.9. These results showed a good model fit [82].

**Table 5:** Structural equation fitting index.

χ^2^	df	χ^2^/df	RMSEA	GFI	CFI	NFI
302.336	144	2.100	0.051	0.931	0.978	0.958

Path analysis was conducted to examine the hypotheses by using AMOS 22.0. According to Holmbeck’s [83] method of testing the mediating effect, the mediating effects of generational interaction, optimistic emotions, and psychological resilience were verified. The results are shown in Table 6.

**Table 6:** Pairwise parameter comparisons.

Hypothesis	Path	β	*t*-Value	*p*-Value	Results
H1	Family Travel→Generational Interaction	0.649	13.477	***	Supported
H2	Generation Interactional→Well-being	0.430	8.554	***	Supported
H3	Family Travel→Optimistic Emotion	0.539	8.600	***	Supported
H4	Optimistic Emotion→Well-being	0.217	4.274	***	Supported
H5	Family Travel→Psychological Resilience	0.541	8.925	***	Supported
H6	Psychological Resilience→Well-being	0.187	3.822	***	Supported
H7	Generational Interaction→Optimistic Emotion	0.133	2.211	0.027	Supported
H8	Optimistic Emotion→Psychological Resilience	0.157	2.722	0.006	Supported

Note: ***p < 0.001.

**Fig 2:**
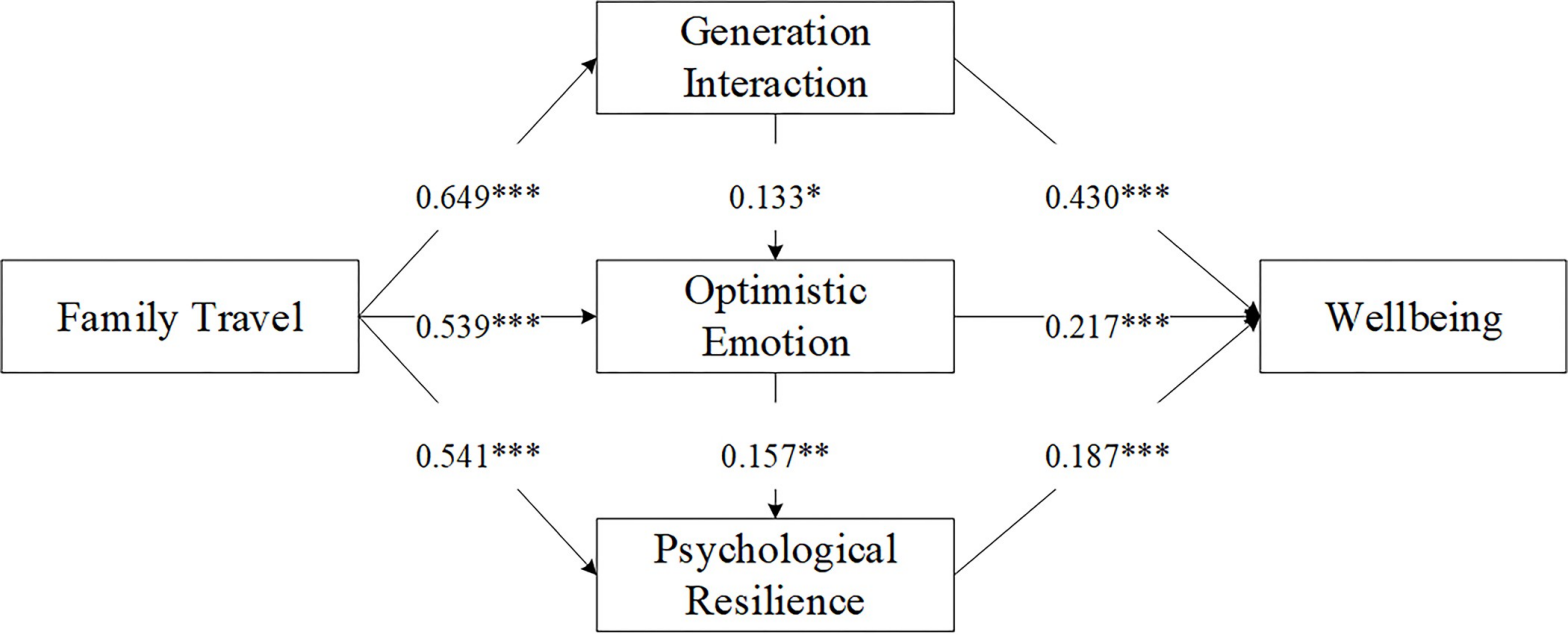
Path analysis results.

In examining the impact of family travel on the well-being of the elderly, the study’s findings confirm that family travel plays a significant role in enhancing generational interaction, optimistic emotions, and psychological resilience, all of which contribute positively to the well-being of older adults. Specifically, path analysis in this study reveals that family travel has a substantial influence on generational interaction (H1), which in turn significantly enhances the well-being of the elderly (H2). Additionally, family travel boosts optimistic emotions (H3), which positively affects well-being (H4). Furthermore, travel increases psychological resilience (H5), contributing to overall well-being (H6). Generational interaction also uplifts optimistic emotions (H7), and these emotions reinforce psychological resilience (H8). Each of these pathways is statistically significant, corroborating the proposed hypotheses. Detailed results are graphically synthesized in Fig 2.

## 5. Conclusion and discussion

### 5.1 Conclusion

With the trend of population aging becoming increasingly pronounced, the pursuit of a contented old age has garnered significant attention. Research has demonstrated that travel can enhance the well-being and life satisfaction of tourists. This study has developed a conceptual framework to examine the effect of progeny–parents family travel on the well-being of elderly individuals, contextualized within the culture of filial piety. Data were collected via questionnaires and analyzed statistically to assess the interconnections among the variables and to test the posited hypotheses. The principal findings of this study are outlined as follows:

1. Progeny–parents family travel markedly boosts the well-being of the elderly. As an engaging social endeavor, family travel exerts a substantial positive influence on the elderly’s psychological state and emotional well-being, thereby amplifying their happiness. This paper corroborates existing research regarding the beneficial impact of tourism activities on elderly well-being, discovering that this effect is a dynamic continuum spanning from pre-travel anticipation to post-travel reflection, encompassing stages of well-being anticipation, experience, and recollection.
2. Intergenerational interaction acts as a mediator in the nexus between progeny–parents family travel and elderly well-being. Echoing the principles of activity theory—which posits that family engagement forms the cornerstone of social existence—the study finds that active participation in family excursions leads to heightened joy for seniors. Throughout these journeys, seniors and their grown children can bolster their mutual affection through positive exchanges, thereby enhancing the seniors’ societal involvement and mitigating the discomforts associated with role transitions, culminating in augmented happiness.
3. Optimistic emotions serve a mediating function between progeny–parents family travel and the well-being of the elderly. The tenets of positive psychology suggest that states such as optimism and resilience are pivotal to individual well-being and life satisfaction. Within the context of progeny–parents family travel, the camaraderie and backing from adult children can harmonize the parent-child bond, invigorate the seniors’ positive emotions, and fortify their well-being. For elders, such travel represents a pursuit of spiritual enrichment and joyous living, fostering not only constructive social bonds but also emotional and spiritual growth.
4. Psychological resilience mirrors the enduring influence of progeny–parents family travel on the well-being of the elderly. This mode of travel has been shown to beneficially impact the elderly’s psychological resilience, thus enhancing their overall well-being. Resilience traits—like hope and tenacity—are crucial internal motivators for a happy and optimistic outlook among seniors. Shifts in the elderly’s social roles can precipitate psychological distress; however, engaging in leisure pursuits such as family travel can offer relief from stress, improve emotional states, and bolster well-being. Seniors who partake in more family travel exhibit heightened self-efficacy and positive psychological traits, endowing them with greater inner strength and elevated life satisfaction.

In concluding, this study dialogues with existing literature by highlighting the unique role of progeny–parents family travel in improving the well-being of the elderly within the framework of Chinese filial piety culture. While previous research has established a general link between tourism and increased well-being, this study delves deeper by elucidating the specific mechanisms at play during family travel that cater to the emotional and psychological needs of the elderly. It underscores the importance of generational interaction, optimistic emotions, and psychological resilience as key mediators of well-being in the context of filial obligations and shared family experiences. This work extends the discourse beyond the immediate joys of travel to consider the enduring effects on elder well-being, aligning with and expanding upon the theories of social engagement and support in gerontology. The findings here not only reaffirm but also enrich the understanding of the transformative potential of shared family experiences on the well-being of older adults, thereby offering new insights for future research and practice in the field of family tourism.

### 5.2 Theoretical contribution

This study offers a groundbreaking perspective on familial tourism by examining its effects on elder well-being through the prism of China’s filial culture. It innovatively links family travel discourse with parental well-being, contextualized by filial duty. Delving into “caring for the elderly” within tourism, it reveals filial piety’s deep influence on travel experiences. The research introduces inventive strategies for bridging generational emotional divides, crafting travel products, and advocating a new model of family travel that resonates with the intertwined values of family, commerce, and culture. By situating the analysis within the Chinese cultural framework, this study challenges the Western-focused narrative on senior travel and enhances our comprehension of Chinese elder tourism behavior. It expands the narrative to include the significant impact of intergenerational travel on elder well-being, thus widening the research scope beyond mere travel decisions to actual family travel experiences. Empirical findings confirm that such shared journeys have a beneficial ripple effect on the well-being of the elderly, enriching the academic conversation and setting a new direction for future exploration in the field.

The real theoretical contribution of this study lies in its comprehensive integration of Activity Theory and Social Support Theory within the unique cultural context of Chinese filial piety, offering a new perspective on elder well-being. First, this research provides a novel framework that connects the well-established Activity and Social Support Theories to the culturally specific construct of filial piety, offering a culturally nuanced understanding of elder well-being. Second, this study extends the scope of Activity Theory by demonstrating how intergenerational family travel acts as a form of active engagement and leisure that promotes elder well-being within a Chinese cultural framework, thus expanding the theory beyond Western contexts. It enhances the Social Support Theory by illustrating how family travel serves as a medium for social support, which is vital for the elderly’s ability to navigate life’s challenges, particularly within societies that emphasize familial obligations. Third, this study introduces an empirical exploration of the emotional and psychological benefits of travel for the elderly, which has been underexplored in previous literature, thus providing evidence for the therapeutic potential of family travel in enhancing life satisfaction and psychological resilience among the elderly. By addressing these points, the study not only fills a significant gap in the existing literature but also sets the stage for future research to build upon its findings, particularly concerning the role of cultural values in shaping the travel experiences and well-being of the elderly.

### 5.3 Practical implications

Family travel, as a leisure lifestyle to enhance the interaction of family members and alleviate the aging crisis, can effectively enrich the lives of the elderly, improve their sense of social existence, help them realize their own value, and positively affect their well-being. The results of this study show that, driven by Chinese filial piety culture, progeny–parents family travel initiated by adult children is of great value when it comes to improving the well-being and travel intentions of the elderly. Based on this, the following implications for the development of the elderly tourism market can be provided:

1. We should pay attention to the influence of family generational interaction and support on improving the well-being of the elderly and effectively exert the positive spillover effect of adult children and other family resources on the tourism of the elderly. Specifically, the development or marketing of tourism products for the elderly should recognize the importance of family, strengthen the publicity and advocacy of traditional filial responsibility, guide and encourage children to pay attention to the maintenance of family emotional relations and the improvement of generational interaction quality, and promote adult children’s care and support for the leisure life of the elderly. Adult children’s company, encouragement, praise, and sharing of tourism experiences can strengthen the elderly’s well-being and positive perceptions of tourism and improve their intention to participate in tourism.
2. In view of the mediating effect of optimistic emotions and psychological resilience on family travel and the well-being of the elderly, it is important to promote the positive mental state and positive psychological capital construction of the elderly and improve their negative tourism cognition to therefore improve the leisure welfare value of the elderly. The decline of physical function, the breakdown of social relations, and the gradual deaths of relatives reduce the scope of social activities of the elderly, and the interactions between the elderly and family members will gradually increase. Adult children, in addition to helping their parents’ basic material livelihoods, should pay more attention to elderly spiritual comfort requirements and provide emotional support (e.g., by travelling with their parents). With the aid of the power of family, effective training, and guiding the elderly to maintain optimistic attitudes, a high level of self-efficacy can be achieved through which the elderly are not afraid of setbacks, display toughness, and have hope for the future, thus helping them actively participate in tourism and other leisure activities and experience happiness in old age.

### 5.4 Limitations and future research

There are some limitations in this study. This study only examined the impact of progeny–parents family travel on the well-being of the elderly. In fact, progeny–parents family travel has a rich impact on the psychology and behavior of the elderly. Still, future research could continue to refine the impact and role of family travel on the psychology and behavior of the elderly. Family travel is a process of intergenerational interaction between parents and adult children. This study only focused on the influence of progeny–parents family travel regarding the parents. Future research could comprehensively examine two-way effects of family travel on the parents and the progeny as well as the spillover effects on other psychological and behavioral effects of the elderly.
